# Disease Activity Is More Associated with IL-1 Than with IL-6 in Patients with Rheumatoid Arthritis

**DOI:** 10.3390/life13010082

**Published:** 2022-12-28

**Authors:** Cristina Almeida-Santiago, Juan Carlos Quevedo-Abeledo, María Vanesa Hernández-Hernández, Antonia de Vera-González, Alejandra González-Delgado, Miguel Ángel González-Gay, Iván Ferraz-Amaro

**Affiliations:** 1 Servicio de Reumatología, Hospital Universitario Dr. Negrín, 35010 Las Palmas de Gran Canaria, Spain; 2 Servicio de Reumatología, Hospital Universitario de Canarias, 38320 Tenerife, Spain; 3 Servicio de Laboratorio Central, Hospital Universitario de Canarias, 38320 Tenerife, Spain; 4 Cardiovascular Pathophysiology and Genomics Research Unit, School of Physiology, Faculty of Health Sciences, University of the Witwatersrand, Johannesburg 2000, South Africa; 5 Division of Rheumatology, Hospital Universitario Marqués de Valdecilla, Universidad de Cantabria, 39011 Santander, Spain; 6 Epidemiology, Genetics and Atherosclerosis Research Group on Systemic Inflammatory Diseases, Hospital Universitario Marqués de Valdecilla, IDIVAL, 39011 Santander, Spain; 7 Departamento de Medicina Interna, Universidad de La Laguna, 38200 Tenerife, Spain

**Keywords:** rheumatoid arthritis, interleukin-6, interleukin-1, interleukin-1 receptor antagonist, disease activity

## Abstract

Interleukin-1 receptor antagonist (IL-1ra) concentration reflects and is proportional to IL-1 production. Both IL-1 and IL-6 are involved in the pathogenesis of rheumatoid arthritis (RA). However, the relationship of serum levels of these two cytokines to each other in RA patients is not well-understood. In this study, our objective was to analyze the possible linear correlation between IL-1ra and IL-6 in patients with RA, and how both are related to the inflammatory activity of the disease. IL-6 and IL-1ra levels were measured in 407 patients with RA. Linear regression and partial correlations were conducted to analyze the relationship between both cytokines, and their association with RA characteristics. No correlation was found between serum levels of IL-6 and IL-1ra (Pearson’s *r* 0.031, *p* = 0.61). However, disease activity and acute phase reactants were positively and significantly associated with both cytokines. Nevertheless, after controlling for covariates, disease activity scores were more strongly associated with IL-1ra compared to IL-6. Circulating IL-6 and IL-1ra do not correlate with each other in RA patients. Although both are associated with disease activity and acute phase reactants, the relationship of disease activity to IL-1ra is greater than that to IL-6.

## 1. Introduction

Cytokines control a wide range of inflammatory processes that are involved in the pathogenesis of rheumatoid arthritis (RA), a chronic inflammatory disease that predominantly involves the joints. An autocrine and paracrine cascade network communication through interleukins (ILs) has a pivotal role in the development of synovitis and perpetuation of the disease. IL-1 and IL-6 are among the most important ILs involved in RA [1]. Anakinra, a recombinant form of the naturally occurring interleukin-1 receptor antagonist (IL-1ra) plus methotrexate was proved to be effective in reducing the signs and symptoms of the disease in patients with inadequate responses to methotrexate alone [2]. This was also the case for a number of biologic agents that blocked the receptor of IL-6 [3].

IL-1 is a critical cytokine that is involved in almost every phase of immune responses, regulating innate immunity and T-cell differentiation [4]. In RA, IL-1 plays a key role in matrix regulation as a potent inducer of matrix metalloproteinases, it is directly responsible for the inhibition of proteoglycan synthesis, and it promotes joint breakdown by attracting inflammatory leucocytes and activating synovial cells [5]. IL-1 comprehends two different cytokines, IL-1α and IL-1β, that act via the same receptor. Furthermore, IL-1ra works as an inhibitor of both cytokines by binding to the receptor [6]. It has been described that IL-1ra concentrations represent IL-1 production, so its levels are proportional to those of IL-1 [7].

IL-6 has a pivotal role in the pathophysiology of RA [8]. This cytokine induces synovitis and joint destruction by provoking neutrophil migration, osteoclast maturation, and vascular endothelial growth factor-stimulated pannus proliferation [9].

Although IL-1 is a potent inducer of IL-6 in RA [10,11,12], the relationship between circulating levels of these proinflammatory cytokines has not been explored before in RA. Due to the link between IL-1 and IL-1ra, and taking into account the recent data shown by our group that revealed that both erythrocyte sedimentation rate (ESR) and disease activity scores were significantly related to higher serum levels of IL-1ra in patients with RA [13], in the present study we analyzed the possible correlation between IL-1ra and IL-6 in patients with RA, and how both are related to the inflammatory activity of the disease. The fact that both ILs were related to each other and differentially to disease activity could have diagnostic and therapeutic implications.

## 2. Materials and Methods

### 2.1. Study Participants

This was a cross-sectional study that encompassed 407 patients with RA. They were recruited consecutively from our clinic outpatient visits. All of them were 18 years old or older and fulfilled the 2010 ACR/EULAR classification criteria [14]. They were periodically followed-up at rheumatology outpatient clinics and had been diagnosed by rheumatologists. Inclusion criteria were: the duration of RA disease was ≥1 year; none of the patients were taking IL-1 or IL-6 receptor inhibitors; patients taking prednisone or an equivalent dose ≤ 10 mg/day. Exclusion criteria were: history of myocardial infarction, angina, stroke; a glomerular filtration rate < 60 mL/min/1.73 m^2^; a history of cancer or any other chronic disease such as hypothyroidism, heart or respiratory diseases, nephrotic syndrome, as well as evidence of active infection. The study protocol was approved by the Institutional Review Committee at Hospital Universitario de Canarias and at Hospital Universitario Doctor Negrín (both in Spain), and all subjects provided informed written consent (approval no. 2019-452-1). All research was performed in accordance with relevant guidelines/regulations and in accordance with the Declaration of Helsinki.

### 2.2. Data Collection, Laboratory Assessments, and Carotid Ultrasound Assessment

Individuals included in the study completed a medication-use questionnaire and underwent a physical examination. Body-mass index—BMI (weight in kilograms divided by the square of the height in meters), waist circumference (measured around and above the hipbones), and systolic and diastolic blood pressure were assessed under standardized conditions. Smoking, diabetes, and hypertension information was obtained from the questionnaire. Disease activity in patients with RA was measured using the Disease Activity Score in 28 joints [15], both using erythrocyte sedimentation rate (ESR)—DAS28-ESR (range 0–9.55) and high-sensitivity C-reactive protein (hsCRP)—DAS28-CRP (range 0–8.47), the Clinical Disease Activity Index (CDAI) [16], and the Simple Disease Activity Index (SDAI) [17]. Cholesterol, triglycerides, and HDL-cholesterol were measured using the enzymatic colorimetric assay. LDL-cholesterol was calculated using the Friedewald formula. ESR was measured through the Westergren method using EDTA (ethylenediamine tetraacetic acid) blood, and hsCRP was assessed by immunoturbidimetry. Human IL-6 was measured by electrochemiluminescence immunoassay method (Roche Diagnostics, Indianapolis, IN, USA). Human IL-1ra was measured using an enzyme-linked immunosorbent assay—ELISA—for its quantitative detection (Biovendor, Brno, Czech Republic). Cytokine assessments were in a concentration range in which the assay produces reliable results. These cytokines were performed for this study, so they were not assessed in our routine clinical follow-up of the patients. IL-6 was measured in the clinical hospital laboratory; this was not the case for IL-1ra.

### 2.3. Statistical Analysis

Demographic and clinical characteristics in patients with RA were described as mean (standard deviation, -SD-) or percentages for categorical variables. For non-normally distributed continuous variables, data were expressed as median and interquartile range (IQR). Normal distribution of variables was tested visually, or using a test for normality when this applied (Kolmogorov–Smirnov). Pearson’s and Spearman correlations were assessed to evaluated the relation of ILs to disease activity parameters. Correlations and corresponding significance were reported. Partial correlation coefficients controlling for covariables were assessed. Partial correlation is a measure of the strength and direction of a linear relationship between two continuous variables whilst controlling for the effect of one or more other covariates or control variables. All the analyses used a 5% two-sided significance level and were performed using Stata software, version 17/SE (StataCorp, College Station, TX, USA). *p*-values < 0.05 were considered statistically significant.

## 3. Results

### 3.1. Demographics and Disease-Related Data

A total of 407 patients with RA were included in this study. Table 1 shows demographic- and disease-related characteristics of the RA patients. The mean age was 56 ± 10 years and 81% of the patients were women. Overall, 22% of the patients were current smokers, 13% had type-2 diabetes, 32% were considered obese (BMI ≥ 30 kg/m^2^), and 34% had hypertension.

### 3.2. Relationship between Demographics and disease data and IL-6 and IL-1ra

The association between demographics and disease-related data with IL-6 and IL-1ra (as dependent variables) is shown in Table 2. While age and sex were not related to both cytokines, BMI and waist circumference were significantly and positively associated with IL-1ra but not IL-6. Regarding CV risk factors, only obese patients disclosed higher and significant serum levels of IL-1ra. hsCRP and ESR, and DAS28-ESR and DAS28-CRP, were significantly associated with higher serum levels of both cytokines. In the case of SDAI and CDAI scores, this association was only significant with IL-1ra (Table 2). Use of any type of conventional DMARD, including hydroxychloroquine, was associated with lower circulating IL-1ra but not IL-6.

### 3.3. Correlation of IL-6 and IL-1ra with Each Other and with Disease Activity Parameters

**Figure 1:**
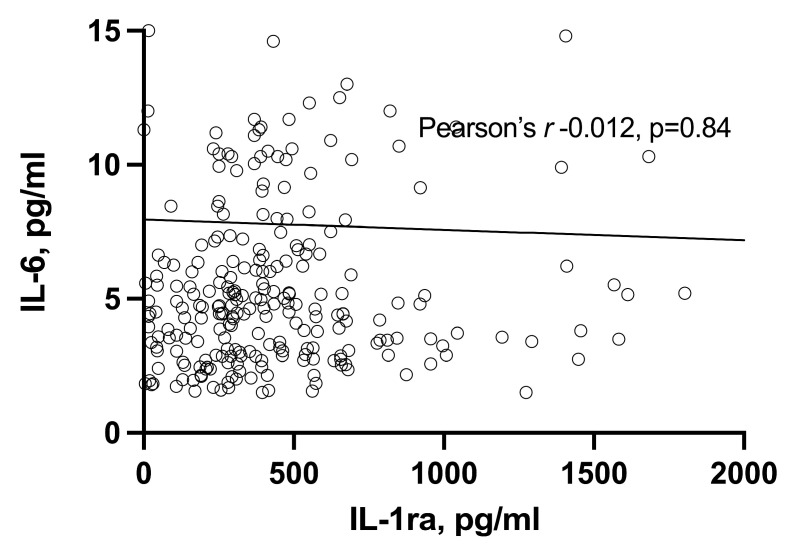
Correlation between IL-1ra and IL-6 serum levels in RA patients.

No correlation was found between serum levels of IL-6 and IL-1ra (Pearson’s *r* —0.012, *p* = 0.84) (Figure 1). This was also the case when patients were categorized according to disease activity, that is, based on disease activity score (remission, low, moderate, and high or very high) (data not shown).

We observed a significant correlation between acute phase reactants and IL-6 and IL-1ra. In this sense, log hsCRP and ESR significantly correlated to both cytokines. Similarly, disease activity scores were also significantly correlated with IL-1ra. However, in the case of IL-6, the correlation was found to be significant for DAS28-CRP but not with other disease activity scores (Table 3).

**Figure 2:**
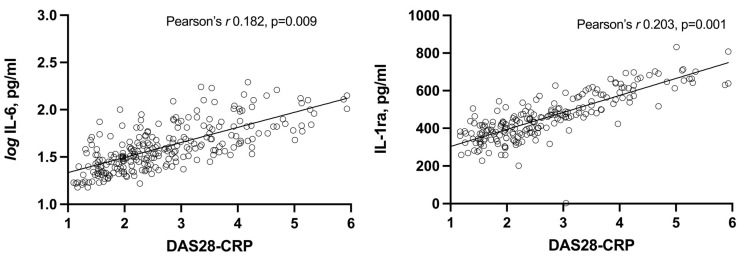
Partial correlations between DAS28-CRP and IL-1ra and IL-6 serum levels in RA patients controlling for covariates.

Several models of partial correlations were assessed to control for covariables. First, the aforementioned relationships of each cytokine to the other were checked. Afterward, ESR and hsCRP were added as covariables; finally, age, gender, and ACPA and RF status were included in the partial correlation model as covariates. In this final model (which included the cytokine not being analyzed, as well as ESR, hsCRP, age, gender, and ACPA and RF status), disease activity scores were significantly correlated with serum levels of IL-1ra. In contrast, in the case of IL-6, a relationship was only found with DAS28-CRP (Table 3). The relation of DAS28-CRP to IL-6 and IL-1ra controlling for covariates is shown in Figure 2.

## 4. Discussion

To our knowledge, our study constitutes the first attempt to assess the relationship between IL-1ra and IL-6 serum levels in a large cohort of RA patients. Both cytokines are key molecules in the pathogenesis of RA. However, we could not find a correlation between them. Furthermore, although both were related to disease activity. This association seemed to be stronger in the case of IL-1ra.

In our work, we measured IL-1ra but not IL-1α and IL-1β. This is due to the fact that serum assessment of α and β types of IL-1 is problematic, since circulating IL-1α is usually absent in serum because it remains in the cytosol of cells, and plasma concentrations of IL-1β are generally below the detection limit of available assays [18]. However, it is well-established that IL-1ra concentrations reflect global IL-1 production, so serum levels of the former have a positive relationship with those of the latter [18].

IL-1 displays several biological activities of IL-6, such as ruling growth and differentiation of cells in the immune and hematopoietic systems, and modulation of acute-phase reactants. In this sense, it is well-established that IL-1 is a potent inducer of IL-6 in fibroblasts, endothelial cells, and keratinocytes [19]. However, it is not clear whether IL-1 has a role in IL-6 gene expression [19]. It has been suggested that cellular responses to IL-1 are mediated by cascades of intracellular events that include the activation of protein kinases involved in the triggering of activated-protein 1, and IkappaB kinase involved in the activation of nuclear factor kappa-light-chain-enhancer of activated B-cells [20]. It is known that combinations of cytokines can have additive, inhibitory, or synergistic effects, and patterns of cytokine production differ under various inflammatory conditions. However, although related to the pathophysiology of RA, they did not show a correlation in the sera of the RA patients included in our study.

Previous work examined the effects of IL-1 on the signaling and action of IL-6 in RA synovial fibroblasts [10]. Pretreatment with IL-1 suppressed Janus kinase signaling by IL-6, and blocked IL-6 induction of tissue inhibitor of metalloprotease expression. These results suggested that proinflammatory cytokines may contribute to pathogenesis by modulating or blocking signal transduction by pleiotropic or anti-inflammatory cytokines. Furthermore, this study identified a molecular basis for IL-1 and IL-6 cross-talk in RA synoviocytes and suggests that, in addition to levels of cytokine expression, modulation of signal transduction also plays a role in regulating cytokine balance in RA. In our study, we did not find a relationship in the serum of both ILs. However, both were associated with disease activity, thus demonstrating their probable pathophysiological role in the disease.

As occurred with other cytokines relevant to the acute phase response such as IL-1, tumor necrosis factor-alpha, and interferon gamma, IL-6 is strongly related with inflammation. In keeping with that, in our study IL-6 showed a correlation with the DAS28-CRP score, which uses hsCRP in its calculation. IL-1ra serum levels showed a correlation with DAS28-CRP score but also with others that do not require the assessment of acute phase reactants. For this reason, we feel that the determination of IL-1ra serum levels may constitute a reliable marker of disease activity in patients with RA.

IL serum levels have been found to be related to several RA-related features. For example, fatigue has been described to be more prominent as serum IL-6 level increases independently of the disease duration and activity [21]. Similarly, in a study of 57 patients with RA in which several cytokines, including IL-1 and 6, were measured, plasma levels of the second were found to correlate to DAS-CRP, CDAI, SDAI, ESR, and CRP [22]. Contrary to the findings of our study, in this work, no relationship was found between IL-1 and the clinical activity parameters of the disease. However, our work has included a much higher number of patients, which represents a more homogeneous RA population with a greater diversity of manifestations and clinical activity of the disease. IL-6 has also been shown to affect lipid metabolism by stimulating hepatic fatty-acid synthesis and adipose-tissue lipolysis to increase cholesterol synthesis while decreasing cholesterol secretion, and to be related to the anemia or osteoporosis that accompanies RA [9]. Similarly, IL-1 inhibition has been found to improve glycemic parameters in RA [23]. Based on all of the above, both ILs, through their relationship with disease activity, or through mechanisms independent of it, probably play a role in the comorbidities present in RA.

We acknowledge several limitations in our work. First, our study is purely clinical. Future works should clarify the relationship between both interleukins at the cellular and in vitro level in patients with RA. These studies may also include IL-1α and IL-1β. Second, we have not recruited controls. For this reason, we cannot compare the serum levels of IL-6 and IL-1ra with healthy population subjects. The pooled estimate of IL-6, in the general population, has been described in a recent meta-analysis as 5.186 pg/mL (95%CI: 4.631, 5.740) [24]. Similarly, IL-1ra has been estimated as 248 (95%CI: 244, 252) pg/mL [25]. However, we did not aim to compare serum levels of both cytokines between patients and controls, but rather to study the association of these cytokines with disease activity. Third, there are probably other cytokines that have not been assessed in our study that may be also related to disease activity in RA patients. Furthermore, the correlation coefficients found in our work can be considered weak. However, it is to be expected that a single IL does not show a strong relationship with disease activity, since the latter represents a wide spectrum of manifestations not only related to inflammation.

## 5. Conclusions

In conclusion, circulating IL-6 and IL-1ra do not correlate with each other in RA patients. Although both are associated with disease activity and acute phase reactants, the relationship of disease activity to IL-1ra is greater than that to IL-6.

## Figures and Tables

**Table 1:** Demographics and disease-related data in RA patients.

	Rheumatoid Arthritis
	(*n* = 407)
Age, years	56 ± 10
Female, *n* (%)	329 (81)
BMI, kg/m^2^	28 ± 5
Waist circumference, cm	97 ± 13
Cardiovascular data	
CV risk factors, *n* (%)	
Current smoker	88 (22)
Obesity	130 (32)
Hypertension	140 (34)
Diabetes Mellitus	53 (13)
Dyslipidemia	190 (47)
Statins, *n* (%)	129 (32)
Disease-related data	
Disease duration, years	8 (4–15)
hsCRP at time of study, mg/L	2.9 (1.4–6.3)
ESR at time of study, mm/1st hour	18 (8–34)
IL-6, pg/mL	4.8 (3.1–7.5)
IL-1ra, pg/mL	472 (248–586)
Rheumatoid factor, *n* (%)	286 (72)
ACPA, *n* (%)	238 (65)
DAS28-ESR	3.2 ± 1.4
DAS28-CRP	2.7 ± 1.1
SDAI	13 (7–20)
CDAI	8 (4–14)
History of extraarticular manifestations, *n* (%)	32 (9)
Erosions, *n* (%)	151 (41)
Current drugs, *n* (%)	
Prednisone	147 (36)
Prednisone doses, mg/day	5 (3–5)
NSAIDs	179 (44)
DMARDs	362 (89)
Methotrexate	306 (75)
Leflunomide	91 (22)
Hydroxychloroquine	44 (11)
Salazopyrin	27 (7)
Anti-TNF therapy	83 (20)
Rituximab	7 (2)
Abatacept	12 (3)
JAK inhibitors	20 (5)
Data represent percentages, mean ± SD, or median (IQR) when data were not normally distributed.	
hsCRP: high-sensitivity C-reactive protein; ACPA: anti-citrullinated protein antibodies; CV: cardiovascular. General population values of IL-6 and IL-1ra have been described as, respectively, 5.186 pg/mL (95%CI 4.631, 5.740) and 248 (95%CI: 244, 252) pg/mL.	
NSAID: nonsteroidal anti-inflammatory drugs; DMARD: disease-modifying antirheumatic drug.	
TNF: tumor necrosis factor; ESR: erythrocyte sedimentation rate.	
BMI: body-mass index; DAS28: Disease Activity Score in 28 joints.	
CDAI: Clinical Disease Activity Index; SDAI: Simple Disease Activity Index.	
IL-6: interleukin-6; IL-1ra: interleukin-1 receptor antagonist.	

**Table 2:** Univariable relation of demographics and disease data to IL-6 and IL-1ra.

	*log* IL-6, pg/mL			IL-1ra, pg/mL
	beta coefficient (95% CI), *p*			
Age, years	0.006 (−0.003–0.2)	0.18	−3 (−7–1)	0.16
Female	−0.1 (−0.3–0.1)	0.39	−7 (−117–104)	0.90
BMI, kg/m^2^	0.004 (−0.01–0.02)	0.61	**18 (10–26)**	**<0.001**
Waist circumference, cm	0.004 (−0.003–0.01)	0.31	**6 (3–10)**	**<0.001**
Cardiovascular data				
CV risk factors				
Current smoker	0.2 (−0.02–0.4)	0.080	−45 (−144–54)	0.37
Obesity	0.09 (−0.09–0.3)	0.31	**145 (57–234)**	**0.001**
Hypertension	0.1 (−0.04–0.3)	0.13	−2 (−88–84)	0.96
Diabetes Mellitus	−0.1 (−0.4–0.2)	0.45	108 (−14–229)	0.082
Dyslipidemia	**0.2 (0.01–0.4)**	**0.035**	−29 (−112–54)	0.49
Statins	−0.07 (−0.3–0.1)	0.46	24 (−65–113)	0.60
Disease-related data				
Disease duration, years	0.005 (−0.004–0.01)	0.29	**−5 (−9–−0.7)**	**0.024**
*log* hsCRP, mg/L	**0.2 (0.08–0.2)**	**<0.001**	**44 (8–80)**	**0.015**
ESR, mm/1st hour	**0.008 (0.004–0.01)**	**<0.001**	**3 (0.7–5)**	**0.010**
Rheumatoid factor	−0.09 (−0.3–0.1)	0.41	−86 (−182–10)	0.079
ACPA	0.01 (−0.2–0.2)	0.90	−60 (−160–40)	0.24
DAS28-ESR	0.05 (−0.01–0.1)	0.13	**61 (30–92)**	**<0.001**
Remission	ref.	-	ref.	-
Low activity	−0.05 (−0.3–0.2)	0.66	14 (−101–129)	0.81
Moderate and high activity	**0.2 (0.009–0.4)**	**0.041**	**152 (61–244)**	**0.001**
DAS28-CRP	**0.09 (0.01–0.2)**	**0.023**	**67 (30–104)**	**<0.001**
Remission	ref.	-	ref.	-
Low activity	0.2 (−0.07–0.4)	0.17	35 (−81–151)	0.56
Moderate and high activity	**0.3 (0.07–0.5)**	**0.008**	**35 (−81–152)**	**0.001**
SDAI	0.003 (−0.002–0.008)	0.19	**3 (0.2–5)**	**0.036**
CDAI	0.007 (−0.004–0.02)	0.20	**7 (2–12)**	**0.005**
History of extraarticular manifestations	**0.4 (0.1–0.7)**	**0.007**	−10 (−169–148)	0.90
Erosions	0.03 (−0.2–0.2)	0.78	−53 (−142–36)	0.24
Current drugs				
Prednisone	0.08 (−0.1–0.3)	0.37	−32 (−118–55)	0.47
Prednisone doses, mg/day	0.01 (−0.03–0.05)	0.53	−12 (−29–5)	0.16
NSAIDs	0.03 (−0.1–0.2)	0.71	−19 (−103–64)	0.65
DMARDs	−0.001 (−0.3–0.3)	0.99	**−154 (−298–−11)**	**0.035**
Methotrexate	0.04 (−0.2–0.2)	0.69	−56 (−155–43)	0.27
Leflunomide	0.1 (−0.07–0.3)	0.20	−35 (−131–62)	0.48
Hydroxychloroquine	0.07 (−0.2–0.3)	0.57	**−137 (−261–−12)**	**0.032**
Salazopyrin	**0.3 (0.02–0.6)**	**0.036**	−101 (−255–52)	0.19
Anti-TNF therapy	0.09 (−0.1–0.3)	0.42	−7 (−111–96)	0.89
Rituximab	−0.6 (−1.6–0.5)	0.27	−85 (−463–294)	0.66
Abatacept	0.4 (−0.1–0.8)	0.12	−19 (−261–223)	0.88
JAK inhibitors	−0.2 (−0.6–0.1)	0.20	−116 (−298–66)	0.21
Log IL-6 and IL-1ra are the dependent variables in this analysis. Significant *p*-values are depicted in bold.				
CV: cardiovascular; hsCRP: high-sensitivity C-reactive protein; ESR: erythrocyte sedimentation rate; ACPA: anti-citrullinated protein antibodies. PCR is natural-log-transformed. JAK: Janus kinase.				
NSAID: nonsteroidal anti-inflammatory drugs; DMARD: disease-modifying antirheumatic drug.				
TNF: tumor necrosis factor; BMI: body-mass index; DAS28: Disease Activity Score in 28 joints.				
CDAI: Clinical Disease Activity Index; SDAI: Simple Disease Activity Index.				

**Table 3:** Correlation between disease activity parameters and IL-6 and IL-1ra controlling for covariables.

	*log* IL-6, pg/mL		IL-1ra, pg/mL	
	***r***	*p*	*r*	*p*
Correlation				
*log* hsCRP, mg/L	**0.242**	**<0.001**	**0.134**	**0.015**
ESR, mm/1st hour	**0.243**	**<0.002**	**0.155**	**0.010**
DAS28-ESR	0.091	0.133	**0.213**	**<0.001**
DAS28-CRP	**0.136**	**0.024**	**0.195**	**<0.001**
SDAI	0.078	0.19	**0.117**	**0.036**
CDAI	0.077	0.20	**0.157**	**0.005**
Partial correlations				
	Controlled by IL-1ra		Controlled by IL-6	
*log* hsCRP, mg/L	**0.236**	**<0.001**	0.112	0.071
ESR, mm/1st hour	**0.242**	**<0.001**	0.127	0.068
DAS28-ESR	0.076	0.22	**0.210**	**<0.001**
DAS28-CRP	**0.130**	**0.037**	**0.204**	**0.001**
SDAI	0.070	0.264	0.116	0.061
CDAI	0.070	0.261	**0.168**	**0.007**
	Controlled by IL-1ra +hsCRP and ESR		Controlled by IL-6 +hsCRP and ESR	
DAS28-ESR	0.062	0.38	**0.194**	**0.006**
DAS28-CRP	**0.170**	**0.016**	**0.233**	**<0.001**
SDAI	0.075	0.29	**0.190**	**0.007**
CDAI	0.077	0.28	**0.210**	**0.003**
	Controlled by IL-1ra +hsCRP and ESR +age, sex, and ACPA and RF		Controlled by IL-6 +hsCRP and ESR +age, sex, and ACPA and RF	
DAS28-ESR	0.098	0.16	**0.172**	**0.006**
DAS28-CRP	**0.182**	**0.009**	**0.203**	**0.001**
SDAI	0.098	0.16	**0.169**	**0.007**
CDAI	0.098	0.16	**0.182**	**0.004**
hsCRP: high-sensitivity C-reactive protein; ESR: erythrocyte sedimentation rate; IL: interleukin.				
ACPA: anti-citrullinated protein antibodies; RF: rheumatoid factor. PCR is natural-log-transformed.				
CDAI: Clinical Disease Activity Index; SDAI: Simple Disease Activity Index.				
DAS: Disease activity score. Significant values are depicted in bold. *r* refers to Pearson’s coefficient. Correlations between disease activity indices and ILs are controlled/adjusted by the mentioned variables. Significant *p*-values are depicted in bold.				

## Data Availability

The datasets used and/or analyzed in the present study are available from the corresponding author upon request.

## Abbreviations

ACPA: anti-citrullinated protein antibodies, BMI: body mass index, CDAI: Clinical Disease Activity Index, CRP: C-reactive protein, CV: cardiovascular, DAS28: Disease Activity Score in 28 joints, DMARD: disease-modifying antirheumatic drug, EDTA: ethylenediamine tetraacetic acid, ELISA: enzyme-linked immunosorbent assay, ESR: erythrocyte sedimentation rate, HDL: high density lipoprotein, IL-1α: interleukin 1 alfa, IL-1β: interleukin 1 beta, IL-1: interleukin 1, IL-1ra: Interleukin-1 receptor antagonist, IL-6: interleukin 6, IQR: interquartile range, JAK: jakus kinase, LDL: low density lipoprotein, NSAID: nonsteroidal anti-inflammatory drugs, RA: rheumatoid arthritis, RF: rheumatoid factor, SD: standard deviation, SDAI: Simple Disease Activity Index, TNF: tumor necrosis factor.
